# Improvement of Electrophoretic Enantioseparation of Amlodipine by Polybrene

**Published:** 2012

**Authors:** Majid Zandkarimi, Alireza Shafaati, Sayyed Mohsen Foroutan, Charles A. Lucy

**Affiliations:** a *School of Pharmacy, Zabol University of Medical Sciences, Zabol, Iran. *; b *School of Pharmacy, Shaheed Beheshti University of Medical Sciences, Tehran, Iran. *; c *Department of Chemistry, University of Alberta, Canada. *

**Keywords:** Capillary electrophoresis, Capillary coating, Chiral separation, Amlodipine, Polybrene

## Abstract

In chiral and non-chiral electrophoretic resolution of basic drugs, adsorption of analytes to negatively charged capillary wall could lead to poor repeatability of migration time and peak area. In addition, chiral resolutions of basic drugs are commonly performed in low pH buffers. Therefore, longer analysis time due to suppression of electroosmotic flow (EOF) is another dilemma. In this work the improvement effect of polybrene (PB), a cationic polymer, on chiral separation of a model basic drug, amlodipine (AML), was investigated. PB both as a semi-permanent coating agent and as an additive in the running buffer was utilized. Better results were obtained with PB as a buffer additive. Compare to untreated bare silica without using PB in running buffer, addition of 0.0005% PB buffer decreased analysis time downed to 3 folds; efficiency improved up to 5 folds; limit of detection (LOD) and limit of quantification (LOQ) downed to 8 folds and within-day migration time and peak area repeatabilities, in terms of relative standard deviations (RSD) downed to 5 and 20 folds, respectively.

## Introduction

About 40% of drugs in use are chiral (1, 2) and in most cases two enantiomers of chiral drugs exhibit different pharmacological, toxicological or pharmacokinetic properties (3). Therefore, development of analytical methods for enantiomer separation for controlling synthesis, enantiomer purity check, and for pharmacodynamic studies is attracting area of research. Thus, different analytical techniques have been applied such as high-performance liquid chromatography (HPLC), thin-layer chromatography (TLC), gas chromatography (GC), supercritical fluid chromatography (SFC) and capillary electrophoresis (CE).

CE has matured to a powerful technique, especially for analytical enantioseparations. This is primarily due to high efficiency and high flexibility with regard to analytes and to the separation conditions, as well as the low consumption of chemicals and solvents. According to a recent discussion forum on the application of CE in the pharmaceutical industry, the major application of the technique is chiral separations (4, 5).

However, CE has some limitations, especially for chiral and non-chiral resolution of basic drugs. Basic analytes are positively charged and electrostatically attracted to the negatively charged capillary walls. This irreproducible adsorption can lead to migration time and peak area variations (6), and consequently to lower precision. Peak tailing can also mask impurity peaks and may also deteriorate resolution of enantiomers in which two analytes migrate closely after each other. Furthermore, analyte adsorption could raise limit of detection (LOD) and limit of quantification (LOQ) of analysis. 

There are several strategies to avoid undesirable interactions of the analytes with the capillary wall. These include: use of high salt concentration, extremes of pH, buffer additives, and coated capillaries (6). Different approaches have been applied for coating of capillaries: covalently/ cross-linked polymer (7), adsorbed cationic polymers (8-11), adsorbed nonionic polymers (12) and adsorbed surfactants (13-17). Dynamic coatings are more attractive compared to covalently bonded polymer coatings, due to their simplicity, versatility, and low cost. The use of dynamically coated capillaries allows for the rapid, precise, and reproducible separation of moderate to strong basic analytes (pK_a_ > 5) at pH 2.5 (18).

**Figure 1:**
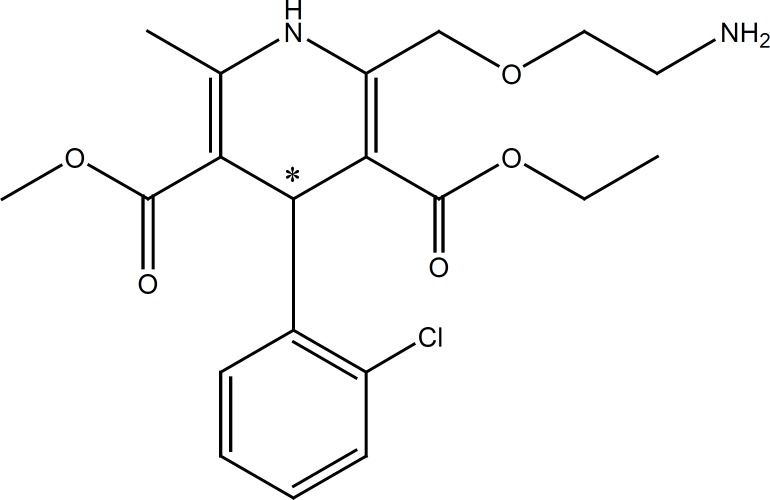
AML structure , pk_a _8.6 (18).

In this work, the effect of a cationic polymer, polybrene (PB), on the improvement of chiral resolution of a model basic drug, amlodipine (AML), was studied. AML (Figure 1) is a potent dihydropyridine calcium antagonist used for the treatment of hypertension and angina pectoris. Only the S (-) isomer of the drug exerts vasodilating action (19).

## Experimental

All solutions were prepared in Nanopure 18 MΩ ultrapure water (Barnstead, Chicago, IL). Sodium phosphate buffer pH 2.5 was prepared from sodium phosphate monobasic monohydrate (EM sciences, Fort Washington, PA), and adjusting the pH with orthophosphoric acid (BDH, Darmstadt, Germany). PB with molecular weight of ~4000-6000 was used as received from Aldrich (Milwaukee, WI). *α*-cyclodextrin; *α*-CD (sigma, Louis, MO), *β*-cyclodextrin; *β*-CD (sigma, Louis, MO), *γ*-cyclodextrin; *γ*-CD (Amaizo, Hammond, Indiana), and hydroxypropyl *β*-cyclodextrin; HP-*β*CD (Cerestar; Hammond, IN) were used as received. Concentration of 10 mM of each cyclodextrin was dissolved in 50 mM sodium phosphate buffer. A solution of 1 mM mesityl oxide (Aldrich) dissolved in water, was used as the neutral EOF marker. Racemic AML and internal standard, racemic verapamil (VER) were provided by Minoo Co. (Tehran, Iran). S-AML was a kind gift from Cipla (Mumbai, India). Racemic AML, S-AML and VER solutions were prepared in water and used throughout the experiments. 

*CE system*

Experiments were performed on a HP^3D^CE capillary electrophoresis system (Hewlett-Packard, Palo Alto, CA, USA) equipped with a diode array detector. CE Chemstation software (Version A.06.01; Hewlett-Packard) was used for control and data acquisition. The data acquisition rate was 6 Hz, and the rise time was 0.2 s. DAD was set at 200 or 254 nm. Untreated fused-silica capillaries (Polymicro Technologies, Phoenix, AZ, USA) with a total length of 45.5 cm (37.0 cm to the detector), an inner diameter of 50 µm, and an outer diameter of 360 µm were used. The capillary was thermostated at 25°C. Samples injection was performed hydrodynamically by applying 50 mbar of pressure for 3s.

Each new capillary was pretreated by rinsing at high pressure (950 mbar) with 0.1 M NaOH for 10 min and with water for 10 min. A Corning model 445-pH meter (Corning Inc., Corning, NY, USA) was employed to adjust the pH of the running buffer. Separations were performed at 20 kV. Depending on the conditions, normal or reverse polarities were applied.

*EOF measurements*

The neutral marker, mesityl oxide (1 mM in water) was injected into the capillary using hydrodynamic injection (50 mbar) for 3s. The electroosmotic mobility (µ_EOF_) was calculated using Equation 1.

$\mu_{\mathrm{EOF}}=\frac{L_{d}\times L_{t}}{\mathrm{MT}\times V}$ (Equation 1)

Where L_t _and L_d _are the total length of the capillary (45.5cm) and the capillary length to the detector (37.0 cm), respectively, MT is the migration time of the neutral marker (in seconds), and V is the applied voltage (in volts).

*Capillary coating*

All of the rinsing procedures performed at 950 mbar. New capillaries were pretreated by rinsing at high pressure (950 mbar) with 0.1 M NaOH for 10 min and with water for 10 min, sequentially. After that, capillaries were rinsed for 3 min with 1% w/v PB in water. Then, capillaries were rinsed for another 10 min with water followed by running buffer for 3 min.

*Coating stability*

The stability of the PB coatings was indirectly evaluated by monitoring the EOF as a function of time. The stability studies were performed in two ways. In the first method, or hydrodynamic rinsing, the coated capillary was flushed with different solutions at a constant pressure (950 mbar) for various times. The EOF mobility was then determined as described above. In the second method, or successive injections (15), a series of runs using 50 mM sodium phosphate buffer containing 10 mM HP-*β*CD with applying -20 kV voltage were conducted. Between runs different washing cycles were applied. Along with, EOF was measured by using mesityl oxide.

*Sample and internal standard preparation*

Standards of 100 ppm of racemic-AML and S-AML were prepared in nanopure water. VER was selected as internal standard since it migrates close to the AML enantiomers.

All samples solutions were spiked with a fixed concentration (20 ppm) of VER.

## Results and Discussion

AML is a basic drug (pK_a_ 8.6) with the primary amine group (20). AML, like other basic drugs acquires positive charge in low pH buffers (21) and has the tendency to adsorb on the bare silica. Thus, modification of capillary wall by PB was considered.

Different strategies have been employed for coating of capillaries by PB. Yeh and coworkers (22) employed 0.1% w/v PB in buffer followed by overnight rinsing of capillaries by 0.2% w/v PB. In another approach, Grob and coworkers (23) utilized 0.001% PB as buffer additive in non-aqueous capillary electrophoresis. Katayama and coworkers (24) modified capillaries by successive multiple ionic polymer layer (SMIL) coating. In this process, anionic polymer, dextran sulfate has adsorbed at the inner wall of capillary by sandwiching PB between dextran sulfate and the capillary wall which resulted in noncovalent adsorption of PB and dextran sulfate layers and a stable coating. The same authors (25) reported an adsorption of an additional PB layer to obtain cationic capillary coatings that possessed a reversed EOF and was demonstrated its application for CE-MS.

**Figure. 2:**
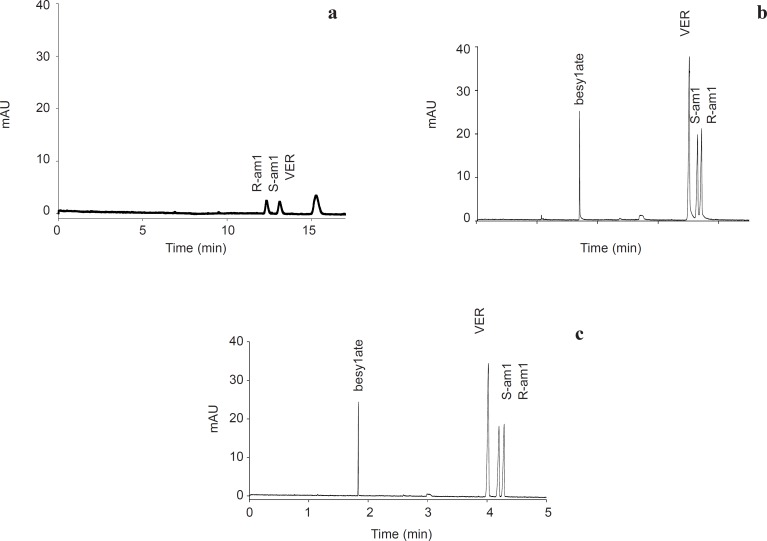
Chiral separation of AML in. a: bare silica. b:semi-permanent coated capillary and c: bare silica using PB 0.005% w/v as buffer additive. Experimental conditions: running buffer 50 mM sodium phosphate( pH 2.5) containin 10 mM HP-*β*CD; wavelength, 200 nm; running voltage +20 kV in (a) and -20 kV in (b &c)) ; sample, AML besylate 100 ppm, VER 20 ppm; other condition as detailed in experimental section

*Resolution of AML in bare silica*

Initially, chiral resolution of AML in bare silica was performed. On the basis of a report published by Owens *et al.* (26) a sodium phosphate buffer containing HP-*β*CD, adjusted to pH 2.5 was employed as background electrolyte. Table 1 shows within day (n = 10) repeatability of migration times, efficiencies, resolution and corrected peak areas in terms of relative standard deviations (RSD). As shown in Table 1, repeatability of efficiencies and corrected peak area ratios in uncoated capillary are poor. These drawbacks could be explained by variable residual low level EOF that may be present even at low pH and/or irreproducible adsorption (6) of AML on the residual silanols. Negligible EOF at pH 2.5 and subsequence longer migration time of the drug causes greater longitude diffusion; this could be another reason for lower efficiencies in bare silica. 

The LOD and the LOQ were estimated as three and ten times the signal-to-noise ratios, respectively. The LOD and LOQ of AML enantiomers in uncoated capillaries were 25 and 80 ppm, respectively.

As shown in Figure 2a slower migrating S-AML in bare silica indicates a stronger interaction between this enantiomer and the selector, which lead to slower movement of this enantiomer toward detector. Same migration order was reported by Owens *et al.* using HP-*β*CD as a neutral selector (26).

**Table 1:** Within-day repeatability in bare silica capillary

	**MT (min)**		**N**		**Rs**	***A***_R_ *** × MT***_VER_	***A***_s_ *** × MT***_VER_	**R/S**
	**R**	**S**	**R**	**S**		***A***_VER_ *** × MT***_R_	***A***_VER_ *** × MT***_s_	
Within-day	12.9	13.7	0.3×10^5^	0.3×10^5 ^	2.1	0.55	0.50	1.082
repeatability ^a^	(3.51)^b^	(3.93)	(38.1)	(44.5)	(17.0)	(21.60)	(19.99)	(5.71)

Experimental conditions: as in Figure 2a.^a^Within-day repeatability: results from 10 experiments performed on the same day for migration times (MT), theoretical number of plates(N), resolution (Rs) and peak areas (A) of R-AML (R) and S-AML (S).^b^ In parentheses %RSD.

*Resolution of AML in semi-permanent coated capillaries*

**Figure 3:**
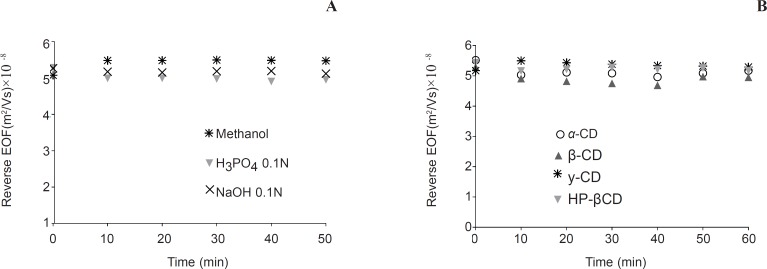
The effect of hydrodynamic rinsing at 950 mbar on EOF of semi-permanent coated capillaries. a: rinsing with washing materials b: rinsing by 10 mM concentration of neutral cyclodextrins. Experimental conditions: running buffer, 50 mM sodium phosphate( pH 2.5) containing 10 mM HP-*β*CD; running voltage, -20 kV; 254 nm; neutral marker, 1 mM mesityl oxide. The coating procedure is as described in experimental section. After coating the capillaries were flushed with the desired washing materials and EOF was measured. Between runs capillaries rinsed 1 min with running buffer

 Capillaries were coated with PB solution under the conditions described previously. PB strongly adsorbed onto the inner capillary surface due to electrostatic attraction between this cationic polymer and the anionic silica (15). Due to altering of capillary charge to positive, direction of the EOF reversed to anodic. AML enantiomers under strong reversed EOF in PB coated capillaries move toward the anode. Therefore, detection could be achieved in reversed polarity. In the coated capillaries, investigating of the coating stability is a priority. A practical approach for doing this is monitoring of EOF (14). Stability of PB coated capillaries evaluated by hydrodynamic rinsing with phosphoric acid, sodium hydroxide, and methanol. Results in Fig. 3a indicate excellent stability of the coating. Therefore, between runs these solutions can be used as cleaning materials.

For enantioresolution, coating should be also stable when cyclodextrins are added to running buffer. Figure 3b shows that PB coated capillaries are stable enough when aqueous solutions of cyclodextrins were used for rinsing the capillary.

**Figure. 4:**
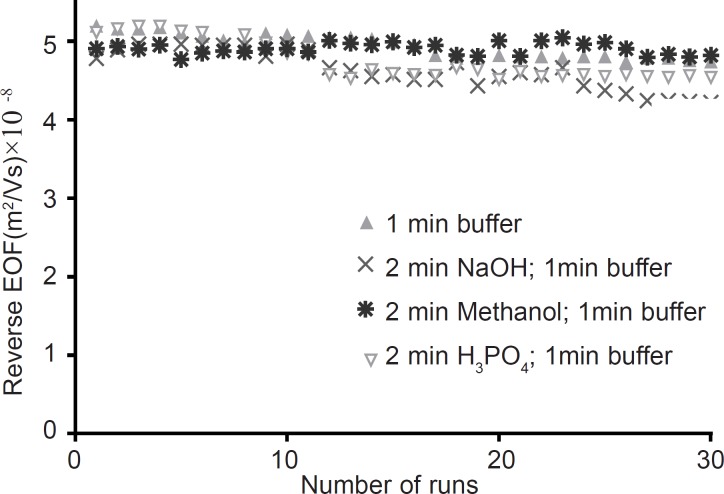
Stability of PB coating layer as a function of the number of runs. Between runs different washing procedures was applied. Experimental conditions: running buffer 50 mM sodium phosphate (PH=2.5) containing 10 mM HP-*β*CD; Running voltage -20 kV; neutral marker1, mM mesityl oxide 245 nm; run time 7 min. The coating procedure is as described in experimental section. Between runs the capillaries were flushed with different washing procedures

The second study of coating stability was performed by 30 successive separations as detailed in experimental section. Results in Figure 4 demonstrate that RSD of EOF measurements are 3.1, 5.4, 5.2 and 1.5 percent for between run rinsing with “running buffer”; “sodium hydroxide followed by running buffer”; “phosphoric acid followed by running buffer”; and “methanol followed by running buffer”, respectively. Therefore “methanol followed by running buffer” flushing suggested as a superior between run rinsing protocol. The stability of PB coated capillaries in this work is superior to the stability of PB coated capillaries reported by Katayama (24).

In the coated capillaries, due to opposite direction of EOF and electrophoretic mobility of AML enantiomers, the more tightly interacted S-enantiomer reached faster to the detector (Figure 2b). Therefore, compare to bare silica migration order reversed. 

One of the advantages of strong reversed EOF in the coated capillary is the ability of resolution of AML counter-ion, besylate, with the drug peak in a single run. Furthermore, compare to untreated bare silica, LOD downed from 25 ppm to 3 ppm and LOQ downed from 80 ppm to 10 ppm. Another advantage of strong reversed EOF is decreasing analysis time up to 3 folds.

**Table 2:** Within -day and between-day repeatability in PB coated capillary

	**MT (min)**		**N**		**Rs**	***A***_R_ *** × MT***_VER_	***A***_s_ *** × MT***_VER_	**R/S**
	**R**	**S**	**R**	**S**		***A***_VER_ *** × MT***_R_	***A***_VER_ *** × MT***_s_	
Within-day repeatability ^a^	3.76 (1.72 )^b^	3.70 (1.68)	1.1×10^5^ (13.9)	1.2×10^5^ (7.1)	1.5 (2.6)	0.50 (1.09)	0.45 (0.83)	1.090 (0.80)
Between-day repeatability ^c^	3.95 (4.82)	3.87 (4.72)	1.1×10^5^ (11.4)	1.2×10^5^ (6.1)	1.6 (6.5)	0.49 (1.29)	0.45 (1.01)	1.089 (0.93)

Experimental conditions: as in Figure 2b.^a^ and ^b ^as in Table 1.^c^ Between-day repeatability: results from 6 experiments performed on 6 days

Table 2 shows within-day and between-day repeatability in PB coated capillary. Although relatively good results in term of migration times, efficiencies, resolution, and corrected peak area repeatability were obtained, but as shown in Figure 2b tailing hump is a major problem. This problem previously reported with using of other cationic polymer (27) and indicates that some interactions still remained at the coated surface. Although enantiomeric ratio of 1.00 should be obtained for a racemic compound, but due to tailing, results in Table 2 demonstrate a drift in R/S ratio. 

**Figure 5:**
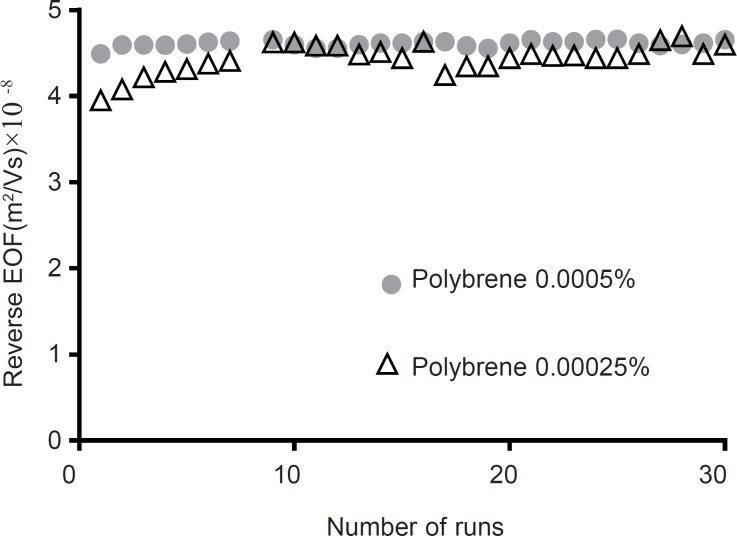
Stability of the EOF in bare silica using PB as buffer additive.

*PB as a buffer additive*

To improving peak tailing, PB was used as a buffer additive. It is known that even low concentrations of PB could reverse the electroosmotic flow (28). As starting point, a concentration of 0.00025% w/v was examined. As illustrated in Figure 5 at this concentration, reverse EOF was observed, however repeatability of EOF were more than 3.8% RSD. At higher concentration of PB (0.0005% w/v) RSD of repeatability of EOF downed to 0.8% RSD in 30 runs. Same as capillary coating, using PB as buffer additive, downs LOD and LOQ up to 8 folds and reduces analysis time more than 3 folds. Compare to coating the capillary, using PB as buffer additive resulted in better efficiency of the separation in terms of plate numbers (Table 3). In addition, peak shape was improved (Figure 2c).

**Table 3:** Within -day and between-day repeatability in bare silica capillary using PB 0.0005%w/v as buffer additive.

	**MT (min)**		**N**		**Rs**	***A***_R_ *** × MT***_VER_	***A***_s_ *** × MT***_VER_	**R/S**
	**R**	**S**	**R**	**S**		***A***_VER_ *** × MT***_R_	***A***_VER_ *** × MT***_s_	
Within-day repeatability ^a^	4.15 (0.78)^b^	4.07 (0.76)	1.6×10^5^ (12.2)	1.6×10^5^ (5.8)	2.0 (4.1)	0.44 (1.26)	0.44 (1.04)	1.007 (0.62)
Between-day repeatability ^c^	4.26 (2.66)	4.17 (2.61)	1.5×10^5^ (13.7)	1.6×10^5^ (10.2)	2.0 (5.1)	0.44 (1.38)	0.44 (1.02)	1.009 (0.84)

## Conclusions

Both using PB as buffer additive and semi-permanent coating agent improve detection limit, efficiency and repeatability of AML chiral resolution. In addition, PB increases speed of the analysis by generating strong reversed EOF. Also, due to strong reverse EOF, simultaneous resolution of AML and besylate was achievable. Although results demonstrate high stability of PB semi-permanent coating but peak tailing for both enantiomers of AML was observed. Therefore, PB as buffer additive selected as a superior approach for improvement of chiral analysis of AML.
